# Behavioral Symptoms May Correlate With the Load and Spatial Location of Tubers and With Radial Migration Lines in Tuberous Sclerosis Complex

**DOI:** 10.3389/fneur.2021.673583

**Published:** 2021-10-22

**Authors:** Rony Cohen, Jacob Genizi, Liora Korenrich

**Affiliations:** ^1^Department of Pediatric Neurology, Schneider Children's Medical Center of Israel, Petah Tikva, Israel; ^2^NF1 and Other Neurocutaneous Disorders Clinic, Schneider Children's Medical Center of Israel, Petah Tikva, Israel; ^3^Sackler Faculty of Medicine, Tel Aviv University, Tel Aviv, Israel; ^4^Pediatric Neurology Unit, Bnai Zion Medical Center, Haifa, Israel; ^5^Department of Imaging, Schneider Children's Medical Center of Israel, Petah Tikva, Israel

**Keywords:** tuberous sclerosis complex, behavioral symptoms, cortical tubers, radial migration lines, Strengths and Difficulties Questionnaire (SDQ)

## Abstract

**Objective:** Tuberous sclerosis complex (TSC) is a multisystem neurocutaneous genetic disorder. The clinical manifestations are extensive and include neurological, dermatological, cardiac, ophthalmic, nephrological, and neuropsychiatric manifestations. The prediction and pathophysiology of neuropsychiatric disorders such as emotional symptoms, conduct problems, hyperactivity, and poor social behavior are poorly understood. The aim of the study was to diagnose neuropsychiatric symptoms in individuals with TSC, and to examine their possible correlations with quantity, magnitude, and spatial location of tubers and radial migration (RM) lines.

**Methods:** The cohort comprised 16 individuals with TSC, aged 5–29 years, with normal or low normal intelligence. The participants or their parents were requested to fill Strengths and Difficulties Questionnaire (SDQ) and the TAND (TSC-associated neuropsychiatric disorders) Checklist for assessment of their neuropsychiatric symptoms. Correlations were examined between these symptoms and the magnitude, quantities, and locations of tubers and white matter RM lines, as identified in T2/FLAIR brain MRI scans.

**Results:** The SDQ score for peer relationship problems showed correlation with the tuber load (*r* = 0.52, *p* < 0.05). Tuber load and learning difficulties correlated significantly in the temporal and parietal area. Mood swings correlated with tubers in the parietal area (*r* = 0.529, *p* < 0.05). RM lines in the temporal area correlated with abnormal total SDQ (*r* = 0.51, *p* < 0.05). Anxiety and extreme shyness were correlated with RM lines in the parietal area, *r* = 0.513, *p* < 0.05 and *r* = 0.593, *p* < 0.05, respectively. Hyperactive/inattention correlated negatively with RM lines in the parietal area (*r* = −707, *p* < 0.01).

**Conclusions:** These observations may lead to future studies for precise localization of neuropsychiatric symptoms, thereby facilitating directed therapy.

## Introduction

Tuberous sclerosis complex (TSC) is a multisystem neurocutaneous genetic disorder caused by mutations in the tumor suppressor genes *TSC1* and *TSC2*, located on chromosomes 9 and 16, respectively. The protein products of *TSC1* and *TSC2* (hamartin and tuberin) function together within the cell and have an inhibitory effect on the mammalian target of rapamycin (mTOR), a protein kinase that influences cell growth and division, and on the synthesis of proteins and other cell components (1). About two-thirds of the occurrences of TSC are sporadic. The clinical manifestations are extensive, including neurological, dermatological, cardiac, ophthalmic, nephrological, and neuropsychiatric (2). These manifestations vary between patients and between genetic mutations, and the expression is age-dependent. About 85% of individuals with TSC exhibit neuropsychiatric manifestations, which include behavioral, psychiatric, intellectual, academic, neuropsychological, and psychosocial impairments (3). Genetic predisposition with *TSC2* mutations (4) and early-onset seizures such as infantile spasm are associated with increased risks of neurodevelopmental and cognitive problems, including autism spectrum disorder (ASD) (5). Structural brain abnormalities such as a higher number of dysplastic lesions on MRI were also shown to predict adverse mental and clinical outcomes (6). The predictors and the etiology of TSC, of behavioral difficulties such as anxiety, depressed mood, learning difficulties, attention deficit hyperactivity disorder (ADHD), and psychosis, have been less investigated. Our aim was to diagnose the neuropsychiatric symptoms in individuals with TSC and to examine their possible correlations with the quantity, magnitude, and spatial location of tubers and radial migration (RM) lines.

## Methods

### The Participants and Brain MRI Evaluation

All patients with TSC who attended our institute between May 2019 and March 2020 with normal or low normal intelligence and brain MRI performed <1 year before evaluation were included. The clinical diagnosis of TSC was made by a senior pediatric neurologist based on the 2012 International Tuberous Sclerosis Complex Consensus Group (7). Individuals with TSC who had motor or moderate–severe intellectual disabilities or intractable epilepsy were excluded. The local ethics committee approved the study. Signed informed consent was obtained from the parents or guardians of each participant before enrollment. The most recent T2-weighted fluid-attenuated inversion recovery (T2-FLAIR) brain MRI scans (<1 year before) of the participants were evaluated by a senior pediatric neuroradiologist. Tubers and white matter RM lines were identified, measured, and classified by their characteristics, location, and size.

### Evaluation of Neuropsychiatric Symptoms

The neuropsychiatric profile was evaluated using the Diagnostic and Statistical Manual of Mental Disorders (DSM-V), in addition to the TAND (TSC-associated neuropsychiatric disorders) Checklist that was translated to Hebrew. This checklist is generally used during meetings with a neurologist in order to assess patients' problems and to help guide a conversation between the clinician and the family or the person with TSC. It consists mainly of a series of YES/NO items in six domains: behavioral, psychiatric, intellectual, academic, neuropsychological, and psychosocial.

The Strengths and Difficulties Questionnaire (SDQ) was used to evaluate children's behavioral and attentional problems. It is an internationally accepted behavioral screening questionnaire for 3- to 16-year-olds (8). Brann et al. (9) support its use also for adolescents (aged 12–17 years) and young adults (aged 18–25 years). We used the American norms. The SDQ consists of 25 items, each rated on a three-point Likert scale (0 = not true, 1 = somewhat true, 2 = true). The items are scored according to five subscales: emotional symptoms (scored from 0 to 5), behavioral problems (from 0 to 3), hyperactivity/inattention (from 0 to 5), peer problems (from 0 to 3), and prosocial behavior (from 6 to 10). The scores for the first four scales are summed to a total difficulty score ranging from 0 to 40, which indicates the likelihood of a psychiatric disorder. In the current study, patients, and/or their parents filled out the questionnaires. If there were differences between their answers, the abnormal results were considered. The results were compared with values in the general population, as reported in the official SDQ site (American norms, https://www.sdqinfo.org).

### Statistical Analysis

All statistical analyses were conducted using IBM-SPSS v.27 (IBM-SPSS, Armonk, NY, USA). Associations were calculated for the scores of the SDQ elements with the numbers and areas of tubers and with RM lines using Spearman's correlation. A *p* ≤ 0.05 was considered significant.

## Results

### Clinical Characteristics

The study group consisted of 16 patients with TSC, aged 5–29 years (mean age = 13 years). Nine (56%) were males. Eleven patients (68%) had epilepsy, and seizures were controlled in 90% of them with oxcarbazepine, carbamazepine, lacosamide, levetiracetam, and valproic acid. Five patients (31%) had a history of infantile spasm, which was treated with vigabatrin. All patients had subependymal nodules (SEN) and one patient had subependymal giant cell astrocytoma (SEGA) (Table 1).

**Table 1:** Demographic and clinical characteristics of 16 individuals with tuberous sclerosis complex.

**Characteristic**	**Number or number (%)**
Male	9 (56%)
Mean age at the brain MRI evaluation, years	13
SEGA	1 (6%)
SEN	16 (100%)
Epilepsy	11 (68%)
Infantile seizures	5 (31%)
Seizure control (of those with seizures)	10 (90%)

### Quantity, Magnitude, and Spatial Location of Tubers

In total, the patients had 273 tubers. The mean number of tubers per patient was 17.2, the minimum number was 2, and the maximum was 39. The range of cyst-like cortical structures detected per patient was 0–22. Fourteen (5.1%) tubers were calcified; none of these were in patients younger than 8 years. The overall quantity, magnitude, and spatial location of the tubers are summarized in Table 2. The mean overall area of the tuber load was 18.0 cm^3^. More tubers were found on the left than on the right hemisphere (mean area = 9.9 vs. 8.5 cm^3^). The area of tubers on the frontal area was the highest (mean area = 6.1 cm^3^), followed by the parietal (4.2 cm^3^), occipital (4.0 cm^3^), temporal (2.9 cm^3^), and the cerebellum areas (0.3 cm^3^). The mean number of RM lines per patient was 7.2 and the range was 1–15. A mean number of 2.1 RM lines was smaller than 0.5 cm^3^, and a mean number of 5.2 was larger than 0.5 cm^3^. The mean areas of RM lines were almost equal on the right and left hemispheres: 3.31 and 3.8 cm^3^, respectively. The mean areas of the RM lines were the largest on the frontal (2.81 cm^3^) and parietal areas (2.4 cm^3)^, followed by the occipital (1.0 cm^3^), temporal (0.94 cm^3^), and the cerebellum areas (0.1 cm^3^).

**Table 2:** Characteristics of the tubers and radial migration (RM) lines in individuals with tuberous sclerosis complex.

	**Mean**	**Minimum**	**Maximum**
No. tubers per patient	17.2	2	39
Cystic tubers only	2.3	0	22
Tuber area (cm^3^): Right	8.5	1	25
Tuber area (cm^3^): Left	9.9	1	24
Tuber area (cm^3^): Frontal	6.1	0	15
Tuber area (cm^3^): Parietal	4.2	0	9
Tuber area (cm^3^): Temporal	2.9	0	8
Tuber area (cm^3^): Occipital	4.0	0	10
Tuber area (cm^3^): Cerebellum	0.3	0	2
Tuber load (cm^3^)	18.0	1.5	53
No. RM lines per patients	7.2	1	15
Area of RM lines (cm^3^): Right	3.3	0	8
Area of RM lines (cm^3^): Left	3.8	0	11
Area of RM lines (cm^3^): Frontal	2.8	0	9
Area of RM lines (cm^3^): Parietal	2.4	0	5
Area of RM lines (cm^3^): Temporal	0.9	0	5
Area of RM lines (cm^3^): Occipital	1.0	0	3
Area of RM lines (cm^3^): Cerebellum	0.1	0	1
RM lines <0.5 cm^3^	2.1	0	13
RM lines >0.5 cm^3^	5.1	0	14

### The TAND Checklist and Correlations With Tubers and RM Lines

Psychiatric diagnoses were made according to the DSM-V criteria and were collected by the TAND Checklist. Fourteen (87%) had psychiatric disorders. ADHD, diagnosed in nine (57%) patients, was the most common diagnosis, followed by learning disabilities in eight (50%). Anxiety disorder was diagnosed in three patients, schizophrenia in two, major depressive disorder in two, obsessive compulsive disorder in one, and ASD in one patient. Behaviors of concern were reported in all but one patient (93%). For 10 (63%), the concern was regarding emotional behavior such as anxiety and depressive mood. Nine reported hyperactive behavior, 7 reported aggressive behavior, 7 peer problems, 4 eating problems, and 19 had sleeping problems. The correlations of the tubers and radial migration lines with the TAND Checklist are presented in Table 3.

**Table 3:** Correlations of tubers and radial migration lines with scores on the TAND Checklist.

		**Tuber load**	**No. tubers**	**Tuber area**	**Tuber area**	**Tuber area**	**Tuber area**	**Tuber area**	**Area of RML**	**Area of RML**	**Area of RML**	**Area of RML**	**RML**
		**Tot**	**Per patient**	**RT (cm^3^)**	**LT (cm^3^)**	**Parietal (cm^3^)**	**Temporal (cm^3^)**	**Cerebellum (cm^3^)**	**LT (cm^3^)**	**Frontal (cm^3^)**	**Parietal (cm^3^)**	**Cerebellum (cm^3^)**	**>0.5cm^3^**
Anxiety	Correlation coefficient	0.054	0.027	0.123	0.095	0.428	0.041	0.478	0.164	−0.014	0.513^*^	0.258	−0.095
	Sig. (2-tailed)	0.842	0.92	0.651	0.726	0.098	0.879	0.061	0.543	0.959	0.042	0.334	0.725
	*N*	16	16	16	16	16	16	16	16	16	16	16	16
Extremely	Correlation coefficient	−0.188	−0.235	−0.173	0.255	0.255	−0.08	0.069	0.221	−0.112	0.593^*^	−0.149	−0.142
Shy	Sig. (2-tailed)	0.486	0.348	0.521	0.34	0.34	0.789	0.8	0.41	0.678	0.015	0.562	0.601
	*N*	16	16	16	16	16	16	16	16	16	16	16	16
Mood	Correlation coefficient	0.068	0.027	0.096	0.529^*^	0.529^*^	0.195	0.06	−0.207	−0.407	0.308	0.228	−0.371
Swing	Sig. (2-tailed)	0.801	0.92	0.723	0.035	0.035	0.489	0.825	0.442	0.118	0.245	0.396	0.157
	*N*	16	16	16	16	16	16	16	16	16	16	16	16
ADHD	Correlation coefficient	−0.25	−0.298	−0.252	0.175	0.175	−0.16	−0.138	0.506^*^	0.530^*^	0	0.149	0.520^*^
	Sig. (2-tailed)	0.349	0.262	0.347	0.516	0.516	0.555	0.081	0.046	0.035	1	0.582	0.039
	*N*	16	16	16	16	16	16	16	16	16	16	16	16
Sleep	Correlation coefficient	0.365	0.33	0.279	0.283	0.283	0.46	0.612^*^	0.175	0.285	0.267	0.537^*^	0.507^*^
Problem	Sig. (2-tailed)	0.165	0.211	0.295	0.283	0.283	0.073	0.012	0.516	0.284	0.318	0.032	0.045
	*N*	16	16	16	16	16	16	16	16	16	16	16	16
Learning	Correlation coefficient	0.589^*^	0.611^*^	0.545^*^	0.566^**^	0.566^**^	0.566^*^	0.179	0.11	0.32	0.333	0.258	0.068
Difficulties	Sig. (2-tailed)	0.021	0.012	0.029	0.022	0.022	0.021	0.506	0.686	0.227	0.207	0.334	0.045
	*N*	16	16	16	16	16	16	16	16	16	16	16	16

* *p <0.05 and*

** *p < 0.01. ADHD, attention deficit hyperactivity disorder*.

Learning difficulties were correlated with the number of tubers per patient (*r* = 0.611, *p* < 0.05) and the tuber load (*r* = 0.569, *p* < 0.05). Tubers in the right and left hemispheres were correlated with learning difficulties (*r* = 0.54, *p* < 0.05, and *r* = 0.57, *p* < 0.01, respectively); the correlation coefficient relating to the left hemisphere was highly significant. Learning problems were also correlated with tuber area in the parietal (*r* = 0.54, *p* < 0.01) and temporal areas (*r* = 0.55, *p* < 0.05). The presence of RM lines in the right hemisphere was correlated with learning difficulties (*r* = 0.12, *p* < 0.05).

ADHD was negatively correlated with RM lines in the left hemisphere (*r* = −0.50, *p* < 0.05) and in the frontal area (*r* = −0.53, *p* < 0.05) and with RM line area >0.5 cm^3^ (*r* = −0.52, *p* < 0.05).

Sleep problems were correlated with tuber area in the cerebellum (*r* = 0.61, *p* < 0.05) and with the area of the RM lines in the right hemisphere (*r* = 0.54, *p* < 0.05) and in the cerebellum (*r* = 0.537, *p* < 0.01). RM lines with an area >0.5 cm^3^ were correlated with sleep problems (*r* = 0.50, *p* < 0.05).

Mood swings correlated with tubers in the parietal area (*r* = 0.529, *p* < 0.05). Anxiety and extreme shyness were correlated with RM lines in the parietal area (*r* = 0.513, *p* < 0.05, and *r* = 0.593, *p* < 0.05, respectively).

### SDQ and Correlation With Tubers and RM Lines

**Figure 1:**
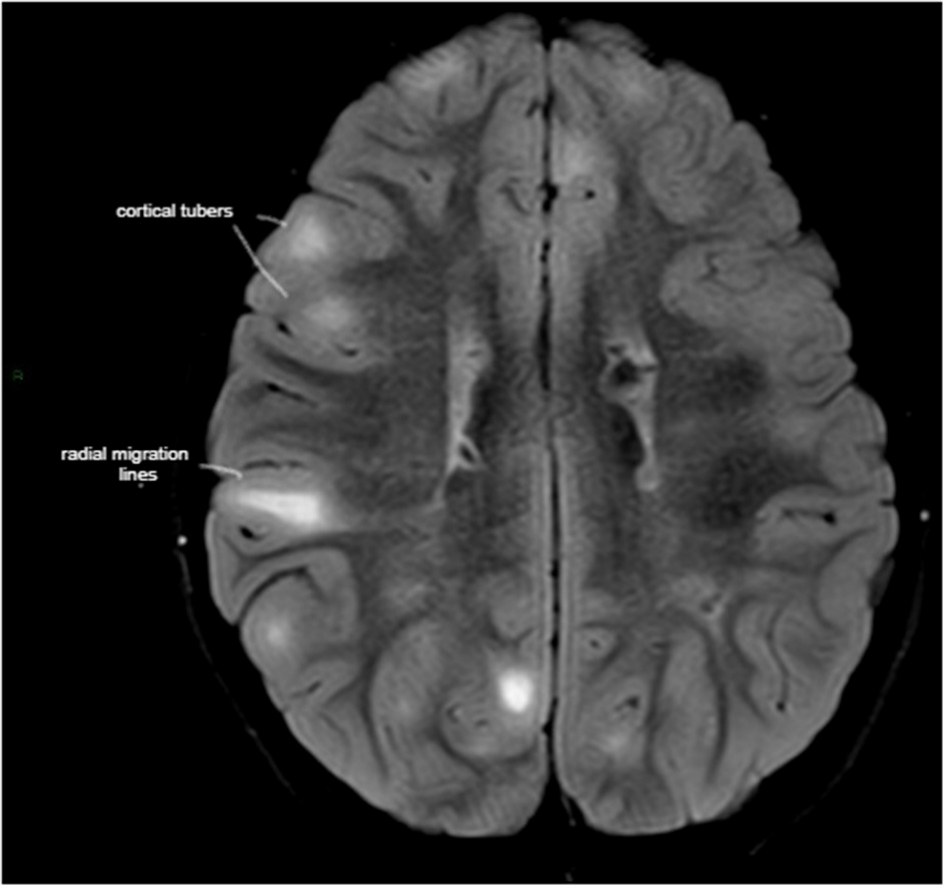
Axial T2-weighted fluid-attenuated inversion recovery (T2-FLAIR) MRI demonstrating high tuber load and radial migration (RM) white lines in a 7-year-old boy with tuber sclerosis complex, normal intelligence, peer relationship problems, and hyperactive/inattention, which correlated negatively with RM lines in the parietal area.

Half of the participants (8/16) scored in the abnormal range of the SDQ for total difficulty. The data elicited from the respondents were tested for normality using the Kolmogorov–Smirnov statistic. The mean (SD) score for total SDQ was significantly higher than that in a normative population [14.44 (*SD* = 7.14), *p* < 0.001]. The mean score for peer problems was also significantly higher, with a mean of 3.5 (*SD* = 2.06, *p* < 0.001). The mean scores for emotional symptoms, conduct problems, hyperactivity, and prosocial behavior were outside the norm, but without statistical significance (Table 4). One of the two patients with SEGA had abnormal SDQ scores in the hyperactive/inattention domain and in prosocial behavior. Both patients with SEGA had abnormal SDQ scores regarding peer problems and also total SDQ scores. Of the 11 patients with epilepsy, two had abnormal emotional SDQ, three had abnormal conduct SDQ scores, five had abnormal hyperactive/inattention SDQ scores, six had abnormal SDQ scores regarding peer problems (Figure 1), and seven had abnormal prosocial behavior scores. Five patients with epilepsy had abnormal total SDQ scores. Two of four patients with a history of infantile spasm had abnormal scores on the emotional, hyperactive/inattention, and conduct domains of SDQ. Three of the four patients had abnormal prosocial behavior scores, and all four had abnormal peer problem behavior. All four of the patients with a history of infantile spasm had abnormal total SDQ scores. Table 5 presents the correlations between the SDQ scores and the tubers and RM lines. The SDQ score for peer relationship problems correlated with the tuber load (*r* = 0.52, *p* < 0.05). The SDQ score of hyperactive/inattention correlated negatively with RM lines in the parietal area (*r* = −707, *p* < 0.01). RM lines in the temporal area correlated with abnormal total SDQ (*r* = 0.51, *p* < 0.05).

**Table 4:** Scores of individuals with tuberous sclerosis complex on the Strength and Difficulties Questionnaire (SDQ).

	**Emotional**	**Behavioral**	**Peer relationship**	**ADHD**	**Prosocial**	**Total**
	**problems**	**problems**	**problems**		**behavior**	**SDQ**
Mean	3.56	2.88	3.50	4.75	6.63	14.44
SD	2.68	2.58	2.06	2.38	2.60	7.14
*P*-value	0.043	0.07	<0.001	0.02	0.006	<0.001

**Table 5:** Correlations of tubers and radial migration (RM) lines with scores on the Strength and Difficulties Questionnaire (SDQ) in individuals with tuberous sclerosis complex.

		**Tuber load**	**Area of RM lines (cm^**3**^):**	**Area of RM lines (cm^**3**^):**
**SDQ domain**			**Parietal**	**Temporal**
Emotional	Correlation coefficient	0.218	−0.149	0.222
Problems	Sig. (2-tailed)	0.417	0.581	0.409
	*N*	16	16	16
Behavioral	Correlation coefficient	0.119	−0.489	0.135
Problems	Sig. (2-tailed)	0.662	0.055	0.619
	*N*	16	16	16
Peer relationship	Correlation coefficient	0.520^*^	−0.048	0.337
Problems	Sig. (2-tailed)	0.039	0.86	0.202
	*N*	16	16	16
Hyperactive/	Correlation coefficient	0.199	−0.707^**^	0.222
Inattention	Sig. (2-tailed)	0.46	0.002	0.409
	*N*	16	16	16
Prosocial	Correlation coefficient	−0.42	0.129	0.122
Behavior	Sig. (2-tailed)	0.105	0.634	0.653
	*N*	16	16	16
Total	Correlation coefficient	−0.005	−0.052	0.511^*^
	Sig. (2-tailed)	0.985	0.85	0.043
	*N*	16	16	16

* *p < 0.05 and*

** *p < 0.01*.

## Discussion

Neuropsychiatric features of TSC present in up to 90% of individuals with TSC (10) and cause great burden to patients and their families (11). Due to the presumed associations of the development of intellectual disability with early and severe epilepsy, efforts have been invested to detecting abnormal background activity in individuals with TSC by routine EEG. The goal is to promote the early initiation of antiepileptic drugs, even before seizures appear (12). In contrast, the prediction, the pathophysiology, and the treatment of neuropsychiatric disorders, such as emotional symptoms, conduct problems, hyperactivity, and poor social behavior, are poorly understood. In this study, we examined the associations of these parameters with cortical tubers and white matter abnormalities. We assessed behavioral problems using the SDQ, a validated questionnaire, and the TAND Checklist, which was designed for the routine examination of individuals with TSC. Total SDQ was significantly higher in our patients with TSC than that in a normative population.

Cortical tubers are focal malformations that consist of various cellular abnormalities, including astrogliosis, dysmorphic neurons, and immature glial cells (13). These tubers are very common in TSC and present in at least 80% of affected patients (14). The wide age range suggests that the associations found are not related to age. The association of tuber load with intellectual disabilities is well-established (6, 14). However, whether this association is due to the severe epilepsy caused by a high tuber load or due to the tuber load itself remains unclear. According to Gipson et al., patients with self-injurious behavior-associated TSC have higher frequencies of tubers in quadrants other than the left posterior neuroanatomical region (15). We found that tuber load and its localization correlate with certain neuropsychiatric symptoms. The SDQ domain for peer relation problems was correlated with cortical tuber load in this study; in some studies, the latter correlated with ASD (16, 17), but others failed to find a correlation (18). Since, in our study, epilepsy was controlled and cognition was normal, the results support the possibility that the tubers interrupt the brain structure and affect peer relation problems directly. Learning difficulties were associated with tuber load in the left hemisphere, mainly in the parietal but also in the temporal area—areas involved in language processing. This is consistent with previous studies (6, 19, 20) that showed associations of significantly high densities of tubers in the inferior parietal lobe and middle temporal lobe with lower IQ scores and learning difficulties. In our study, the somatosensory cortex function correlated with emotional regulation (21), whereas tubers in this location correlated with shyness and mood disorder. Sleep problems correlated with tubers in the cerebellum and also in the right hemisphere. The cerebellum is involved in sleep stage-dependent activity, and its malfunctions can lead to changes in the sleep–wake cycle, resulting in sleep disorders (22). The right hemisphere mediates vigilance and might also affect sleep.

White matter abnormalities have emerged as another important mechanism for brain dysfunction in TSC. Such abnormalities are associated with an overall decrease in measures of functional connectivity, such as a reduced interhemispheric synchrony, between the different regions of the brain (23, 24). The latter appears critical for a variety of brain symptoms of TSC, including intellectual disability, autism, epilepsy, and other psychiatric and behavioral disorders (25). RM lines refer to linear bands seen on MRI radiating from the periventricular white matter to the subcortical region. In our study, RM lines presented more in the frontal area, and their location in the temporal area was correlated with total abnormal SDQ. This supports the possibility that abnormal behavioral symptoms may be related to abnormalities in white matter (25). Specifically, anxiety correlated with RM lines in the parietal area, an area where anxiety increased after stimulation (26). ADHD negatively correlated with RM lines in the left hemisphere and in the frontal area and with RM lines larger than 0.5 cm^3^. Associations have been reported between ADHD and weaker function and structure of prefrontal cortex circuits, especially in the right hemisphere. The prefrontal association cortex plays a crucial role in regulating attention, behavior, and emotion; the right hemisphere is especially associated with behavioral inhibition (27). Both RM lines located in the cerebellum and RM lines larger than 0.5 cm^3^ were found to correlate with sleeping disorders (21). By interfering with the development of neural circuitry, RM lines could impair neurotransmission, thus resulting in deficits in intelligence, communications, and social skills, in seizures (28), and probably also in difficulties in learning, attention, and sleep. The limitation of our study was the relatively small population. Although the genetics analysis was available for only 60% of the patients, since individuals with moderate to severe intellectual disability were excluded, such data were less important. This is the first report of a correlation between behavioral symptoms and RM lines and tuber load in various brain areas. These observations may lead to future studies for precise localization of neuropsychiatric symptoms, thereby facilitating directed therapy.

## Data Availability Statement

The raw data supporting the conclusions of this article will be made available by the authors, without undue reservation.

## Ethics Statement

The studies involving human participants were reviewed and approved by Helsinki Committee, Rabin Medical Center. Written informed consent to participate in this study was provided by the participants' legal guardian/next of kin.

## Author Contributions

RC and JG examined the patients and participated in revising the article. LK revised the brain MRI's and participated in revising the article. All authors contributed to the article and approved the submitted version.

## Conflict of Interest

The authors declare that the research was conducted in the absence of any commercial or financial relationships that could be construed as a potential conflict of interest.

## Publisher's Note

All claims expressed in this article are solely those of the authors and do not necessarily represent those of their affiliated organizations, or those of the publisher, the editors and the reviewers. Any product that may be evaluated in this article, or claim that may be made by its manufacturer, is not guaranteed or endorsed by the publisher.
